# Immune Escape Mechanisms in Non Small Cell Lung Cancer

**DOI:** 10.3390/cancers12123605

**Published:** 2020-12-02

**Authors:** Andrea Anichini, Valentina E. Perotti, Francesco Sgambelluri, Roberta Mortarini

**Affiliations:** Human Tumors Immunobiology Unit, Department of Research, Fondazione IRCCS Istituto Nazionale dei Tumori, Via Venezian 1, 20133 Milan, Italy; valentina.perotti@istitutotumori.mi.it (V.E.P.); francesco.sgambelluri@istitutotumori.mi.it (F.S.); roberta.mortarini@istitutotumori.mi.it (R.M.)

**Keywords:** lung adenocarcinoma, lung squamous cell carcinoma, pre-invasive lesions, carcinoma in situ, immune escape, HLA loss of heterozygosity, immune contexture, neoantigens, dysfunctional T cells, immunotherapy, immune checkpoint blockade

## Abstract

**Simple Summary:**

Immune escape mechanisms have been identified in every step of lung tumorigenesis. A heterogeneous ensemble of immune evasion processes shapes the evolution of precursor lesions towards the invasive phase, contributes to fostering progression from early stage to metastatic disease and defines the main immunological features of specific lung cancer molecular subsets. By disabling distinct phases of the cancer immunity cycle, from antigen presentation to activation of T cell-mediated immunity, and from antigen-specific T cell recruitment to tumor recognition, immune escape in non small cell lung cancer (NSCLC) represents one of the major hurdles to be overcome to improve clinical outcome in patients treated with immunotherapy targeting immune checkpoints.

**Abstract:**

Development of strong immune evasion has been traditionally associated with the late stages of solid tumor progression, since advanced cancers are more likely to have reached the third phase of the immunoediting process. However, by integrating a variety of approaches, evidence for active immune escape mechanisms has been found even in the pre-invasive lesions that later progress to the main NSCLC histotypes. Pre-invasive lesions of adenocarcinoma (LUAD) and of squamous cell carcinoma (LUSC) can show impaired antigen presentation, loss of heterozygosity at the Human Leukocyte Antigen (HLA) region, neoantigen silencing, activation of immune checkpoints, altered TH1/TH2 cytokine ratios, and immune contexture evolution. Analysis of large panels of LUAD vs. LUSC, of early stage NSCLC vs. normal lung tissue, of specific molecular subsets of NSCLC, and of distinct regions within the same tumor, indicates that all these processes of immune escape continue to evolve in the invasive stage of NSCLC, are associated with inter- and intra-tumor heterogeneity, and contribute to resistance to therapy by immune checkpoint blockade (ICB). In this review, we will discuss the most recent evidence on immune escape mechanisms developing from the precursor to invasive stage in NSCLC, and the contribution of immune evasion to resistance to ICB in lung cancer.

## 1. Introduction

According to the principles of the immunoediting theory, the development of immune escape mechanisms in neoplastic lesions allows tumor cells to survive the initial phase of tumor elimination dependent on the activation of anti-tumor immunity [1]. Anti-tumor immunity and immune escape first reach a phase of equilibrium and, as neoplastic progression evolves, immune escape becomes dominant. It seems therefore reasonable to argue that: (a) pre-malignant, precursor lesions of invasive NSCLC, showing immune activation, may also present evidence of some early immune escape mechanisms; (b) the strength and multiplicity of specific escape mechanisms may increase from pre-invasive to early invasive to advanced stage cancers; (c) immune escape mechanisms in pre-invasive lesions may be a driving force for the emergence of dominant clones that will become the invasive tumor. Evidence consistent with these arguments is emerging from a set of recent studies that have addressed the immune structure and complexity of pre-invasive NSCLC lesions and of early stage NSCLC.

In pre-invasive lesions, as well as in invasive squamous and adenocarcinoma subtypes, the selective pressure of the immune system impacts on the HLA system, on the antigen presentation pathway, and on the neoantigen profile of the lesions. Immunoediting in tumors with high tumor mutational burden (TMB) leads to the selection of neoplastic cells characterized by subclonal loss of heterozygosity at the HLA loci (HLA LOH) and to copy number loss or epigenetic silencing of genes containing neoantigenic mutations [2].

Impairment of anti-tumor immunity, developing in early stage lung cancer, emerges from studies focusing on high-dimensional single-cell profiling of the lung adenocarcinoma (LUAD) tumor microenvironment compared to adjacent normal lung tissue. These studies show that the tumor lesions that contain a high frequency of regulatory T cells (Tregs) and of dysfunctional T cells are characterized by exclusion of functional Natural Killer (NK) cells and of immunostimulatory CD141^+^ dendritic cells (DCs), and by presence of immunosuppressive macrophages [3].

Immune escape mechanisms contribute to explaining intrinsic and acquired resistance of NSCLC to immunotherapy targeting immune checkpoints (immune checkpoint blockade, ICB). The efficacy of cancer immunotherapy in NSCLC is hampered by neoantigen loss or intra-tumor heterogeneity for neoantigens, by the development of an immunosuppressive microenvironment secondary to tumor aneuploidy and by constitutive, non-immuno-mediated, tumor PD-L1 expression. Some NSCLC molecular subsets show specific mechanisms that may promote resistance to immunotherapy due to the function of the genetic alterations that they harbor. These include mutations of *EGFR*, fostering an uninflamed microenvironment [4], and of *STK11*/*LKB1*, associated with STING silencing and therefore with suppression of the intracellular DNA sensing pathway leading to type I IFN production [5].

## 2. Lung Cancer Pre-Invasive Lesions: Morphology, Genomic Features, Immune Evolution, and Immune Escape Mechanisms

The 2015 WHO lung cancer classification [6] recognizes four lung cancer pre-invasive lesions: atypical adenomatous hyperplasia (AAH), adenocarcinoma in situ (AIS), squamous cell carcinoma in situ (CIS), and diffuse idiopathic pulmonary neuroendocrine cell hyperplasia. These lesions are thought to progress to lung adenocarcinoma (LUAD), lung squamous cell carcinoma (LUSC), and carcinoid tumors, respectively.

### 2.1. LUAD Pre-Invasive Lesions: Morphological and Genomic Features

Adenocarcinoma evolves in a stepwise fashion from AAH, to AIS, then to minimally invasive adenocarcinoma (MIA), and finally to invasive stage [7,8]. Both precursor lesions of invasive adenocarcinoma (AAH and AIS) show a lepidic pattern of growth, i.e., a cell proliferation of cuboidal to columnar cells along the lining of the alveolar structures, associated with variably atypical nuclei [8]. AAH is usually a small lesion (<0.5 cm) found in peripheral lung showing proliferation of atypical type II pneumocytes [8]. Cellular and structural atypia are more pronounced in AIS, which is in general a larger localized lesion (>5 mm, but less than or equal to 3 cm) containing densely packed atypical cells. MIA is a small lesion (less than or equal to 3 cm in diameter) with a predominant lepidic pattern and an invasive structure smaller than or equal to 5 mm [8]. MIA is usually non-mucinous and is defined as lacking lymphatic, vascular, and pleural invasive components [8].

Multi-region exome sequencing studies [9] have recently confirmed the stepwise evolution of invasive LUAD from precursor lesions. Total mutational burden, reflecting the accumulation of single nucleotide variants (SNVs), progressively increases from AAH to AIS and further to MIA and LUAD [9]. Few somatic copy number alterations (SCNAs) were detected in AAH, but became prevalent in AIS, MIA, and LUAD. Analysis of multifocal lesions at different stages showed higher TMB and higher proportion of clonal mutations in lesions at more advanced stages [9].

### 2.2. Immune Activation in LUAD Pre-Invasive Lesions

Immune activation can be detected at the earliest step of premalignancy, in the AAH lesions, and shows further development alongside transition to subsequent stages. Lymphocyte infiltration increases in premalignant AAH lesions compared to non-tumoral areas and reaches the highest levels in AIS and LUAD [10]. Analysis of CD4, CD8, FOXP3, PD-1, and PD-L1 expression in AAH and LUAD, by immunohistochemistry, provided evidence for T effector and cytotoxic cells and expression of the PD-L1 checkpoint in premalignancy [10]. The average percentage of CD8^+^ T cells correlated with the percentage of progression-associated neoantigens in AAH, but not in AIS/LUAD [10]. Furthermore, AAH lesions with greater neoantigen loads had significantly more infiltrating CD4^+^ T cells and PD-L1-positive cells [10]. Higher levels of CD8^+^ T cells in invasive LUAD, compared to AIS and to MIA, have even been reported by a different study [11]. Deconvolution of gene expression data using the computational tool TIMER has confirmed the evolution in immune composition in the transition from normal lung (NL) tissue to LUAD. A progressive increase in CD4^+^ T lymphocyte infiltration from (NL) to invasive LUAD, and an opposite trend in CD8^+^ T cells, have been detected by such an approach [12]. Similarly, B cell infiltration and tertiary lymphoid structure (TLS) signatures progressively increased from normal lung (NL) to invasive LUAD [12].

### 2.3. Evidence for Immune Escape in LUAD Pre-Invasive Lesions

**Figure 1:**
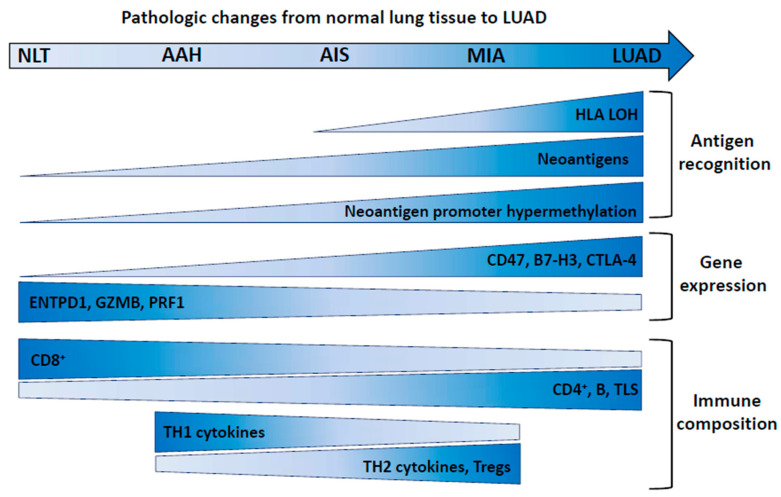
Immune evolution in LUAD pre-invasive lesions. Changes in the indicated parameters along with progression of pre-invasive lesions to LUAD are shown by size and color of the shapes (larger vertical size and darker color: increase). NLT: normal lung tissue; AAH: atypical adenomatous hyperplasia; AIS: adenocarcinoma in situ; MIA: minimally invasive adenocarcinoma; LUAD: lung adenocarcinoma. The indicated evolution of CD8^+^ cells is according to reference [12], while other studies [10,11] report a different trend. Tregs: regulatory T cells; HLA-LOH: loss of heterozygosity at the HLA loci.

The earliest evidence of immune escape mechanisms in LUAD precursor lesions has been documented by Chen et al. [13], who found HLA loss of heterozygosity (LOH) in 3.1% of AIS/MIA compared to 16.7% of LUAD specimens (Figure 1). In a different study [12], LOH at the HLA loci was observed in 7%, 15%, and 33% of AIS, MIA, and LUAD, respectively, but in none of the AAH lesions [12]. Interestingly, these authors also found a progressive increase from AAH to AIS, then to MIA and to LUAD, in predicted major histocompatibility complex (MHC)-I- or MHC-II-associated neoantigens [12]. A larger proportion of genes associated with predicted neoantigens exhibited promoter hypermethylation (>30% CpG methylated) in later stage lesions, suggesting neoantigen depletion in more advanced stage lesions [12].

The evolution of the immune-related gene landscape from normal lung (NL) tissue, through precursor lesions (AAH, AIS, MIA), and to LUAD has been investigated by the Nanostring nCounter pan-cancer immune profiling panel [12]. This approach provided evidence for a progressive increase in the expression of genes involved in negative regulation of immunity [12]. *CD47*, enabling the escape of cancer cells from macrophage-mediated phagocytosis [14], *CD276* (B7-H3) and *CTLA4*, two immune checkpoint molecules involved in inhibition of immune response [15,16], were progressively increased (Figure 1). *ENTPD1* (expressed on tumor-specific T cells), as well as the cytotoxic factors granzyme B (*GZMB*) and perforin 1 (*PRF1*), expressed by T cells and NK cells, showed progressive decrease. TH1 cytokines were reduced in AIS and MIA compared to AAH, while levels of TH2 and Treg cytokines were higher in later stages, consistent with the hypothesis of T cell reprogramming towards pro-tumor function during evolution of preinvasive lesions [12].

### 2.4. LUSC Pre-Invasive Lesions: Morphological and Genomic Features

LUSC evolves through a series of morphological changes promoted by chronic inflammation in the bronchial epithelium [17]. This stepwise process includes basal cell hyperplasia (BCH), squamous metaplasia (SM), grade I–III dysplasia, and carcinoma in situ (CIS). BCH and SM are frequent in smokers and in patients with chronic obstructive pulmonary disease (COPD). While BCH and SM are thought to be reactive and reversible processes, the shift from metaplasia to dysplasia is considered an oncogenic step [17], while dysplasia and CIS are the true premalignant lesions. However, even BCH and SM can harbor genetic abnormalities and are considered potential premalignant lesions [17]. In BCH, ciliated cells and three or more layers of basal cells are present, whereas goblet cells are absent [18]. In SM, columnar ciliated respiratory epithelium is replaced by mature squamous epithelium [18]. Dysplasia (from mild to severe) shows increasing levels of abnormalities (in terms of enlarged cells, epithelial thickness, pleomorphism, and mitoses). In CIS, cells usually show a marked increase in size, can present marked anisocytosis and pleomorphism, and there is no progression of maturation from base to luminal surface [18].

The changes in genomic structure, gene expression, and DNA methylation associated with the transition from precursor lesions to LUSC have recently been elucidated by Teixeira et al. [19]. These authors characterized longitudinally monitored pre-invasive bronchial lesions to decipher differences between progressive and regressive lesions. CIS samples and The Cancer Genome Atlas (TCGA) Research Network LUSC tumors showed a similar mutation burden and copy number profile. Even type and frequency of potential driver mutations (i.e., those previously implicated in lung cancer) and CIS mutational signatures (showing a strong tobacco-associated signal) were in agreement with those previously found in LUSC [19]. However, principal component analysis (PCA) of CIS lesions showed a clear distinction between those that progressed and those that regressed. Regressing lesions clustered with normal epithelium in the analysis of the methylation profile [19]. Five of the top differentially expressed genes (i.e., more expressed in progressive CIS lesions) were previously associated with chromosomal instability [19]. In conclusion, pre-invasive bronchial lesions at the CIS stage share several genomic changes with advanced invasive LUSC, but transcriptomic and epigenetic differences exist between lesions that are benign and those that will progress to cancer.

### 2.5. Concurrent Development of Immunity and Immune Escape in LUSC Pre-Invasive Lesions

**Figure 2:**
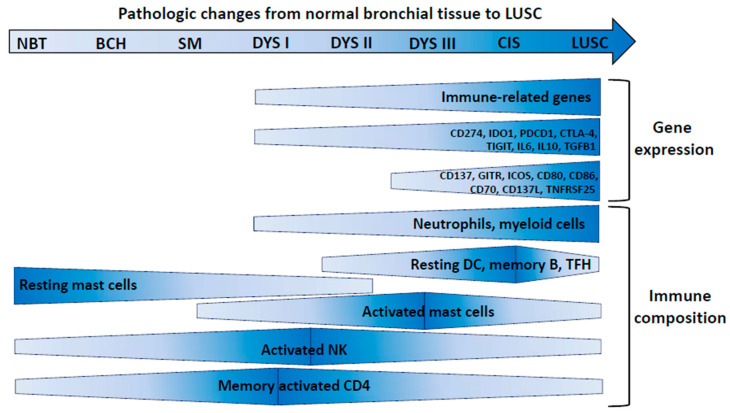
Immune evolution in LUSC pre-invasive lesions. Changes in the indicated parameters along with progression of pre-invasive lesions to LUAD are shown by size and color of the shapes (larger vertical size and darker color: increase). NBT: normal bronchial tissue; BCH: basal cell hyperplasia; SM: squamous metaplasia; DYS I–III: grade I–III dysplasia; CIS: carcinoma in situ; LUSC: lung squamous cell carcinoma. DCs: dendritic cells; TFH: T follicular helper cells; NK: natural killer cells.

Generation of immunity and activation of immune escape can represent early and concurrent events in precursor, pre-invasive bronchial lesions [20]. Lesions encompassing nine morphological stages from normal bronchial epithelium (stage 0) to LUSC (stage 8) were profiled by gene expression analysis [20]. Seven modules of co-expressed genes were identified that showed a specific association with nine morphological stages of progression. Each module was characterized by a distinctive pattern of change: gene expression could in fact progressively increase or decrease at a specific stage along progression towards malignancy. One of the gene modules, named “ascending from high-grade” (since increasing gene expression was observed from moderate dysplasia onwards), contained the highest percentage of immune-related genes. This suggests that strong immune activation occurs from such an advanced stage of premalignancy. Immune-related genes were instead under-represented in the “descending module” (i.e., a module with a linear decrease of gene expression from normal lung to cancer). By deconvolution of gene expression data [20], an increase in myeloid-derived cells, neutrophils and macrophage subtypes, activated T cells (CD4 memory), macrophages (M0), memory B cells, follicular T-helper cells, and dendritic cells was documented in high-grade dysplasia (Figure 2).

A number of Gene Ontology processes, such as the negative regulation of the immune system and antigen processing and presentation, were implicated in all developmental stages. However, genes associated with negative regulation of immunity were significantly downregulated in low-grade lesions, but upregulated in high-grade lesions and LUSC [20], suggesting promotion of immunosuppression in high-grade lesions.

In severe dysplasia, and in subsequent stages, the same authors [20] found higher expression of inhibitory molecules (such as *IDO1*, *PD-L1*, *TIGIT*, *CTLA4*), and of suppressive interleukins (such as *IL10* and *IL6*). However, even stimulatory molecules such as *TNFRSF9*/*CCD137*, *TNFRSF18*/*GITR*, *ICOS*, *CD80*, *CD86*, *CD70*, *TNFSF9*/*CD137L*, and *TNFRSF25* showed increased expression in high-grade dysplasia and even higher levels at the invasive stage [20], indicating concurrent evolution of immune activation and immune suppression. By seven-plex immunofluorescence panels, associated with spatial analysis, CD4^+^ and CD8^+^ T cells showed a transitory increase in high-grade pre-invasive lesions associated with increased densities of myeloid cells, neutrophils, and macrophages in the stroma. In high-grade lesions, a spatial segregation of CD3^+^ lymphocytes from CK^+^ epithelial cells was found. These findings strongly support a model of concurrent development of immunity and immune escape in precursor lesions of LUSC.

### 2.6. Immune Escape in CIS That Progresses to Invasive LUSC

The available evidence suggests that lesions that progress to invasive stage are those where the immunity/escape balance is tilted in favor of immune escape and of immunosuppression, while regressive lesions have a dominant anti-tumor response. In agreement, by a variety of approaches, regressive CIS lesions were shown to contain more lymphocytes, more intra-lesional CD8^+^ cells, and increased levels of genes coding for pro-inflammatory cytokines (*IL2*, *TNF*, *IL12A*, and *IL23A*) compared to CIS progressive lesions [21]. Hierarchical clustering of all the data obtained by different approaches led to the identification of clusters of “cold” lesions and almost all of them progressed to cancer. However, some “hot” lesions also progressed to cancer. Intriguingly, progressing lesions expressed more putative neoantigens [21]. To gain insight into these apparently counterintuitive findings, the authors tested the hypothesis of activation of a dominant immune escape mechanism in progressive “hot” lesions (Table 1). In agreement with this hypothesis, mutations and copy number alterations affecting 62 genes involved in antigen presentation by MHC, antigen processing, and immunomodulation were more frequent in progressive lesions than in regressive lesions [21]. Frequency of loss of heterozygosity (LOH) in the HLA region was similar in progressive and regressive lesions, but progressive lesions showed a striking cluster of hypermethylation in chromosome 6, including the region containing all of the major HLA genes [21]. Additional key differences between progressive and regressive CIS lesions included downregulation of *TNFSC9*/*CD137L* and a higher *CCL27*/*CCR10* ligand:receptor pair ratio in progressive lesions [21].

### 2.7. LUSC Pre-Invasive Lesions Can be Classified into Distinct Immune-Related Subtypes

Bronchial pre-malignant lesions can be classified according to specific patterns of gene expression, which may be variably associated with histological severity and with specific immune processes (Table 1). Beane et al. [26], through definition of co-expressed gene modules and via consensus clustering, identified four molecular subtypes of bronchial pre-malignant lesions: proliferative (27.4% of the investigated lesions), inflammatory (19.5%), secretory (32.1%), and normal-like (21.1%). Genes related to immune activation and inflammatory response were more expressed in the inflammatory and secretory subsets. Genes related to interferon signaling and antigen presentation were more expressed in the proliferative and secretory subset. The normal-like subtype showed the most decreased expression of genes involved in inflammation, regulation of lymphocytes and leukocytes, and antigen processing and presentation pathways. Within the proliferative subtype, progressive pre-malignant lesions showed downregulated expression of genes involved in interferon signaling and antigen processing, compared with regressive biopsies.

## 3. Immune Escape Mechanisms in Early Stage NSCLC

### 3.1. Immune Landscape in Early LUAD

Lavin et al. [3] found that stage I LUAD already has significant alterations in several immune compartments, and early evidence of emerging immune suppression and immune escape (Table 1). In surgically resected early tumors, from treatment-naive patients, high-dimensional single-cell profiling indicated higher immune infiltrate in tumor compared to normal lung (NL), with T cells and mononuclear phagocytes being the most abundant lineages. T and B lymphocytes were present at a higher frequency in the tumor microenvironment compared to the NL, whereas NK cells were reduced in all cases investigated. Immune cells accumulated mainly in the stroma and at the invasive margin around the tumor islets. Tertiary lymphoid structures (TLS) were present near the tumor invasive margin, but absent from NL. Tregs expressing CTLA4, CD39, and ICOS were significantly increased in the tumor lesion, even at early stages [3]. Cytolytic CD8^+^ T cells were significantly reduced in frequency in the tumor compared to NL and blood.

CD8^+^ T cells expressing PD-1 were clonally expanded at the tumor site, preferentially in TLS-enriched tumor lesions. NK cells at the tumor site expressed low levels of granzyme B and CD57, as well as low levels of IFNγ. The CD16^+^ NK cell subset was the most reduced in the tumor compared to NL [3].

Evidence of immune escape, documented by tumors showing reduction of MHC class-I-expressing neoplastic cells, was associated with higher numbers of tumor-infiltrating CD16^+^ NK cells, suggesting NK-dependent immunoediting of MHC-I-positive tumor cells [3]. PD-L1^+^ macrophages accumulated in clusters at the tumor invasive margin and negatively correlated with T cell infiltration in tumor lesions. CD141^+^ DCs, but not CD1c^+^ DCs, were significantly reduced in tumors compared to NL [3].

Collectively, these results indicated that the tumor microenvironment of early LUAD shows profound alterations of several immune compartments, including enrichment for PPARγ^hi^CD64^hi^CD14^hi^IL-6^hi^ macrophages, CD1c^+^ DC, Tregs, and exhausted T cells and depletion of CD141^+^ DCs, CD16^+^ monocytes, NK cells, and granzyme B^+^ effector cells.

### 3.2. Multiple Tumor Subsets in Early LUAD by Immune-Related Gene Classification

The immune landscape of never smoker female patients with early stage LUAD has been recently investigated [23]. From an initial list of 564 literature-retrieved, immune-related genes, eight functional categories in the cancer-immunity cycle were generated. These eight gene modules were differentially expressed in the patients’ panel, allowing for the identification of three major subsets [23]. Subgroup 1 had significantly lower expression of MHC I/II genes, antigen processing machinery, including *B2M*, NK cell activation, and type I IFN production. Subgroup 2 was defective in ubiquitinating enzyme expression, while the type I IFN and TGF-β signaling pathways were significantly downregulated. Subgroup 3 maintained relatively high levels of MHC class II and of other cancer immune gene expression. The immune score (i.e., level of the infiltration of immune cells in tumor tissue) was the highest in subgroup 3 and the lowest in subgroup 1. Subgroup 2 showed low expression of peptide-trimming machinery (ERAPs and CANX) and of MHC class II, while most patients in subgroup 3 showed expression of MHC class II, but low expression of MHC class I [23].

### 3.3. HLA Loss of Heterozygosity in Early NSCLC

The TracerX collaboration [2] addressed the impact of HLA loss of expression in early stage NSCLC (Table 1). Analysis of different NSCLC cohorts indicated a very low frequency of mutations predicted to disrupt antigen presentation or the MHC class I complex (5–8% of the tumors). However, a computational tool (named “loss of heterozygosity in human leukocyte antigen”, LOHHLA) allowed for the determination of HLA allele-specific copy number from sequencing data, indicated HLA LOH in 36/90 (40%) of NSCLCs, where either the maternal or paternal allele was lost, resulting in HLA homozygosity. HLA LOH was more frequent in LUSC (61%) than in LUAD (29%). By multi-region analysis, HLA LOH was found to frequently occur subclonally (i.e., present only in a subset of cancer cells) in both major histotypes, indicating occurrence later in evolution, possibly in response to a shift in favor of immune evasion [2]. No tumors exhibited homozygous deletion of HLA, in agreement with the hypothesis that maintaining one copy of HLA genes may avoid NK-mediated elimination. In tumors with HLA LOH, there was a significant increase in the number of subclonal non-synonymous mutations (i.e., expressed only in a fraction of neoplastic cells), but not in the number of clonal mutations. This finding suggests that HLA LOH may allow for the accumulation of potentially antigenic, but subclonal mutations.

Neoantigen prediction, based on patient-specific non-synonymous mutations, documented the presence of putative neoantigens that could bind the HLA allele lost due to HLA LOH [2]. Tumors exhibiting HLA-LOH showed increased expression of PD-L1, suggesting that HLA LOH may induce immune escape in response to an active immune response. In agreement, the cytolytic activity score (level of expression of *GZMA* and *PRF1*), CD8^+^ and NK infiltrate were increased in tumors with HLA LOH [2]. These authors concluded that HLA LOH, due to its frequency and subclonal nature, occurs as a late event in tumor evolution, as a local, region-specific process, triggered in reaction to an active immune microenvironment, and that ultimately shapes branched tumor evolution.

### 3.4. The Relationship Between Immune Structure, HLA LOH, and Neoantigen Expression in NSCLC

The notion that immune response to an early tumor may act as a selective pressure driving the emergence of immune evasion is supported by a further study from the TRACER X consortium [22]. By immune deconvolution tools, as well by conventional pathology estimates of tumor infiltrating lymphocytes (TIL), levels of immune infiltration in multi-region NSCLC samples were defined. Tumors with uniformly high or low levels of infiltration by 16 different immune subsets were found in each of the two major histotypes, although tumors showing heterogeneous infiltration of distinct regions were also identified [22]. These authors then tested the hypothesis that neoantigen depletion/silencing could occur in tumors/regions with a more pronounced level of immune infiltrate. Indeed, tumors with high/heterogeneous levels of immune infiltration showed a lower proportion of ubiquitously expressed clonal neoantigens (i.e., neoantigens expressed in all neoplastic cells) compared to tumors with low levels of immune infiltration [22]. Tumors without HLA LOH (i.e., potentially retaining full capacity for antigen presentation) and with high/heterogeneous level of immune infiltration, showed depletion of expressed neoantigens. Copy number loss and gene silencing by promoter hypermethylation were found to explain the loss of neoantigens. Furthermore, evidence for pervasive disruption of antigen presentation through HLA LOH (56% of LUAD and 78% of LUSC) was confirmed by this study [22].

### 3.5. A Relationship of Dysfunctional T Cells at Tumor Site with Burden of Clonal Neoantigens

A recent study in stage I–IIIa, untreated NSCLC patients has shown that the tumor mutational burden and the clonal structure of neoantigens are associated with the generation of dysfunctional T cells at the tumor site [35]. By multiparametric flow cytometry, terminally differentiated dysfunctional T cells (TDTs) were identified as CD57^+^PD-1^hi^ CD8^+^ or CD4^+^ lymphocytes with high expression of GZMB and Eomes, while the CD57^−^PD-1^+^ fraction was labeled as dysfunctional (Tdys). Samples with paired whole exome sequencing (WES) and flow cytometry data were subjected to clustering through self-organizing maps (SOMs). By such a computational tool, the clusters correlating with TMB showed a CD45RA^−^PD-1^hi^ phenotype (including CD4 T_dys_, TDT, and TDT_GZMB_ and CD8 T_dys_, TDT, and TDT_Eomes−_ populations). In contrast, clusters that negatively correlated with TMB lacked PD-1 expression and included progenitor-like CD4 and CD8 subsets. The burden of clonal (i.e., expressed in all neoplastic cells), but not subclonal mutations, correlated with an increased frequency of dysfunctional subsets among CD4 and CD8 T cells. TDTs and Tdys contained neoantigen-specific T cells, confirming that T cell exhaustion at the tumor site can result from chronic antigen stimulation. Moreover, progenitor-like subsets of Tdys and of TDTs were clonally related, as documented by T cell receptor, (TCR) sequence analysis. Taken together, these results indicate that progenitor-like subsets of CD4 and CD8 T cells may differentiate into dysfunctional T cell states as a result of an antigen-driven process promoted by the expression of clonal neoantigens in the tumor microenvironment.

## 4. Complexity and Heterogeneity of the Immune Landscape of LUAD and LUSC Subtypes

A large set of studies has revealed a complex and heterogeneous immune landscape of the two main NSCLC histological subtypes [28]. For example, LUSC tumors have been shown to contain twice the amount of Tregs as LUAD lesions, associated with concomitant reduction in T_H1_ and T_H17_ cells, but are enriched in CD8_EMRA_ (CCR7^−^ CD45RA^+^) and in CD8^+^ PD-1^+^ cells [28]. The complexity and multiplicity of LUAD and LUSC immune subsets has been deciphered by two complementary approaches: (a) association of the immune structure with the expression subtypes identified by whole genome gene expression analysis; (b) the definition of “hot” and “cold” tumor clusters by integration of flow cytometry data, immune-related gene expression, and inference of immune cell composition by the deconvolution of RNA-seq data.

### 4.1. Main LUAD and LUSC Expression Subtypes are Associated with Distinct Immune Landscapes

Subtypes of NSCLC, defined by distinct gene expression profiles, have been identified by the Cancer Genome Atlas Research Group in LUSC and LUAD [36,37]. Four expression subtypes (primitive, classical, secretory, and basal) have been identified in LUSC and three in LUAD (proximal inflammatory, proximal proliferative, and terminal respiratory unit). Each subtype may express a distinct immune profile [28]. In LUAD, the lowest expression of most immune cells was found in the proximal proliferative subtype. The terminal respiratory unit subtype showed higher levels of DCs, CD56^bright^ NK cells, mast cells and eosinophils, B cells, T_FH_ cells, T_CM_ cells, T_H17_ cells, and CD8^+^ T cells [38] compared to proximal proliferative tumors. The latter subtype showed higher expression of TH1 and TH2 cells, Treg cells, cytotoxic T cells, and NK CD56^dim^ cells. Among LUSC, the classical and secretory subsets showed the lowest and the highest immune cell expressions, for innate and adaptive immunity, respectively [38].

### 4.2. Identification of “Hot” and “Cold” Clusters of Tumors

By t-distributed stochastic neighbor embedding (t-SNE) of flow cytometry data in NSCLC lesions, Lizotte et al. [39] found an immune-rich “hot” cluster, a “cold” cluster, and a third small cluster, characterized by strong granulocytic infiltrate. The “hot” cluster, enriched in LUSC lesions, (while LUADs were similarly distributed in the hot and in the cold clusters), was characterized by strong signals for CD8^+^ T cells, high expression of inhibitory receptors PD-1 and TIM-3 on CD8^+^ T cells, FOXP3^+^ Treg infiltration and expression of PD-L1 on tumor and immune cells. By transcriptomic analysis, the hot cluster showed high expression of genes (as *IFNG*, *STAT1*, *CCR5*, *CXCL9*, *CXCL10*, *CXCL11*, *IDO1*, *PRF1*, *GZMA*, and *HLA-DRA*) contributing to defining the well-known “IFN-γ response signature” [39]. More recently, three immune-related clusters have been identified by Gillette et al. [27] in LUAD by deconvolution of RNA-seq data to infer immune cell composition: a “hot” tumor-enriched (HTE), a “cold” tumor-enriched (CTE), and a normal adjacent tissue (NAT-enriched) cluster. HTE tumors were distinguished from CTE tumors by their stronger signatures for B cells, CD4^+^ and CD8^+^ T cells, dendritic cells, and macrophages. On the other hand, HTE tumors also showed strong signals for Tregs and for immunosuppressive cytokines, such as TGF-β1 and IL-10. The NAT-enriched cluster had immune infiltration signatures that were intermediate between the HTE and CTE subtypes.

### 4.3. Molecular Mechanisms Leading to Neutrophil Recruitment in LUSC

Tumor-associated neutrophils (TANs), thought to exert a pro-tumoral and immunosuppressive function in the lung tumor microenvironment, are more abundant in LUSC than in LUAD, while macrophages show the opposite trend [40]. Recently, the molecular mechanism of enhanced TAN recruitment in LUSC has been elucidated by Mollaoglu et al. [41]. By mouse models, the lineage-defining factors *SOX-2* and *NKX2-1* (where the former suppresses the latter) were shown to inversely regulate the TAN chemoattractant CXCL5, a chemokine whose human ortholog is CXCL6. By the opposite regulation of CXCL5, SOX2 promoted TAN recruitment, while NKX2-1 repressed it. Interestingly, the suppression of *NKX2-1* by *SOX2* induced squamous lung carcinogenesis and, strikingly, the TAN population itself drove such differentiation. Furthermore, the TAN population in these models showed pro-tumoral features including increased ROS activity, elevated expression of genes that block T cell activity, and promoted extracellular matrix (ECM) degradation [41]. Thus, key transcription factors expressed in lung epithelial cells drive recruitment of TANs that in turn promote squamous carcinogenesis.

## 5. Immune Escape Mechanisms Involved in Resistance of NSCLC to Immunotherapy Targeting Immune Checkpoints

### 5.1. Neoantigen Silencing or Loss Associated with Immunotherapy Resistance

Loss of expression of neoantigens recognized by T cells can impact not only on disease evolution, but even on response to immunotherapy (Table 1). As previously mentioned, Rosenthal et al. [22] showed that epigenetic silencing of genes harboring mutations that generate neoantigens is a mechanism of immune escape in NSCLC. Among genes that carry neoantigenic mutations, an 11.4-fold increase in promoter hypermethylation was observed for genes that were not expressed, in comparison to genes that were expressed [22]. The role of neoantigen loss in immunotherapy resistance has been directly addressed in two studies [24,25]. McGranahan et al. [24] investigated the fraction of tumor cells harboring a mutation that leads to the generation of a putative neoantigen. They classified neoantigens as clonal (expressed in all neoplastic cells), or subclonal (expressed only in a fraction of cells) and showed that the immune landscape of the tumor and clinical benefit after immunotherapy in NSCLC depend not only on the neoantigen burden, but even on the neoantigen clonal structure of the tumor. A high and clonal neoantigen burden was associated, in LUAD, with a hot tumor microenvironment characterized by the infiltration of the tumor by neoantigen-specific T cells. In addition, tumors from patients with no durable benefit showed higher neoantigen intra-tumor heterogeneity (ITH), compared to tumors from patients with a durable clinical benefit [24]. Almost every tumor with a low neoantigen subclonal fraction (<5% subclonal) and high mutation burden (≥70, median clonal neoantigens of the cohort) demonstrated durable clinical benefit with anti-PD-1 therapy [24]. Moreover, significantly longer progression-free survival (PFS) was documented in patients whose tumors showed a high clonal neoantigen burden and low neoantigen ITH. Furthermore, the majority of clonal neoantigens were attributed to smoking-induced mutations. In a second study, Anagnostou et al. [25] carried out longitudinal analysis of pre/post immunotherapy lesions from NSCLC patients developing acquired resistance after an initial response. The loss of seven to 18 putative mutation-associated neoantigens was found in the post-therapy resistant tumors. Neoantigen loss occurred through the elimination of tumor subclones (i.e., by immunoediting of the tumor) or through deletion of chromosomal regions and LOH.

### 5.2. Immune “Cold” Landscape and Immunotherapy Resistance in LKB1/STK11-Deficient LUAD

Immunotherapy resistance in NSCLC can be associated with the molecular subtype of the tumor (Table 1). In LUAD, the immune landscape is influenced by co-occurring mutations [31]. LUAD with *KRAS* and *p53* mutations shows a hot, T cell-infiltrated tumor microenvironment, higher TMB, and the expression of several molecules belonging to the immune checkpoint class [31]. In contrast, when KRAS mutations are associated with *STK11*/*LKB1* deficiency, the tumor landscape is cold and associated with reduced PD-L1 expression. Co-mutations of *KRAS* and *CDKN2A/B* show a mixed immune profile [31]. Gillette et al. [27] confirmed an immune-cold structure in *STK1*1-mutated tumors, characterized by the most dramatic downregulation of immune activation with marked reductions in DC, NK, T cell, and macrophage signatures. Pathway enrichment identified neutrophil degranulation to be the signature most strongly associated with *STK11* mutation, although neutrophils did not appear to be either specifically enriched or depleted in *STK11* mutant tumors.

A recent study in mouse models by Koyama et al. [32] has shown that *LKB1* inactivation is associated with neutrophil accumulation in the immune microenvironment and with overproduction of tumor-promoting cytokines. Kitajima et al. [5] provided crucial mechanistic insight into the immune-cold structure of *LKB1*-deficient tumors. They showed that this genetic defect results in silencing of *STING*, leading to loss of the ability to sense cytoplasmic double-strand DNA (dsDNA), a crucial type I IFN activation pathway in the early phases of the immune response. When the pathway is functional, cytoplasmic dsDNA is detected by cGAS, that in turn activates STING through cGAMP. STING then binds to TBK1 and bridges it to IRF3, eventually inducing expression of type I IFNs. Therefore, *LKB*1 deficiency generates a powerful immune escape mechanism that blocks the generation of tumor immunity in the very early stages of the process. Furthermore, *STING* silencing in *LKB1*-deficient tumors was regulated by an epigenetic mechanism. In fact, the *STING* promoter showed evidence of hypermethylation (associated with enhanced DNMT1 activity). Moreover, accumulation of H3K27Me3 levels (due to enhanced EZH2 activity) were also found at the promoter of *STING* [5].

Conversely, Della Corte et al. [42] have recently identified an *STK11*-deficient LUAD subset that presents co-mutations of p53. This subset has a “STING^high^ profile” associated with strong immune-related gene expression. These results indicate that among *LKB1* mutant tumors there is a range of immune profiles determined by distinct co-mutations (i.e., *KRAS* vs. *p53*).

The association of LKB1 deficiency with poor response to PD-1 blockade has been shown in 174 patients with *KRAS* mutant LUAD of the Stand Up To Cancer cohort (SU2C). Objective response rates (ORRs) to PD-1 blockade were 7.4% in *KRAS*/*LKB1* mutant tumors, 35.7% in *KRAS*/*p53* mutant, and 28.6% in *KRAS* only tumors [43]. In the same study, the authors report that the ORRs were 0% vs. 57.1% vs. 18.2%, respectively, in the three KRAS molecular subsets, even among patients enrolled in the CheckMate-057 phase III trial [43]. In addition, in the SU2C cohort, *KRAS*/*LKB1* mutant patients had shorter PFS and overall survival (OS) compared to *KRAS*^MUT^/*STK11*/*LKB1*^WT^ tumors [43].

Other studies [44,45,46] have not confirmed that *LKB1* deficiency is always associated with poor outcome after ICB. The first evidence was obtained by assessment of a subset of patients (429/1274) among those enrolled in the Keynote 042 trial (pembrolizumab vs. chemotherapy for previously untreated, PD-L1-expressing, locally advanced or metastatic NSCLC). This subset had WES data allowing for the identification of those with *STK11* mutations (*n* = 33, 7.69%) and, interestingly, patients with or without *STK11* mutations showed similar ORRs, PFS, and OS [44]. In addition, pembrolizumab resulted in improved outcomes compared to chemotherapy, regardless of *STK11* mutation [44]. Papillon-Cavanagh et al. [45] have assessed the impact of *STK11* mutations in the Flatiron Health Clinico-Genomic Database (CGDB), a real-world data cohort including 2276 patients. These authors found that *STK11* mutations are prognostic rather than predictive and are associated with poor prognosis regardless of treatment [45]. Finally, complete response to nivolumab therapy has been recently described in two patients with LKB1-deficient and *p53*-mutated LUAD [46]. Taken together, these studies suggest that the LKB1 deficiency is not necessarily associated with PD-1 resistance, in agreement with the evidence [42] that such a mutation can be found even in tumors that do not have a STING^low^/immune “cold” profile.

### 5.3. Immune-Cold Structure and Immunotherapy Resistance in EGFR^mut^ LUAD

An association between a specific molecular trait of the tumor and immune-cold microenvironment, that predisposes to resistance to immunotherapy, exists in *EGFR* mutant tumors (Table 1). Chen et al. [33] found that EGFR activation (due to, for example, exon-19 deletions, and L858R mutation) induced constitutive PD-L1 expression. In other words, in *EGFR* mutant tumors, PD-L1 expression is not the result of an immune process (i.e., upregulation induced by IFN-γ produced as result of an ongoing immune response), but is a mechanism of tumor immune escape. Of note, even the presence of *EML4-ALK* fusion products [34] has been shown to promote constitutive expression of PD-L1. In a different study [4], patients with *EGFR* mutation showed a lack of T cell infiltration, a reduced proportion of PD-L1^+^/CD8^+^ TIL, and decreased tumor mutational burden. Analysis of data from four randomized trials also indicated that patients with *EGFR* mutation did not benefit from PD-1/PD-L1 inhibitors compared to patients with wild-type EGFR [4].

### 5.4. Chromosomal-Level Alterations Shape an Immunosuppressive Microenvironment Predisposing to Immunotherapy Resistance

Immune evasion in the tumor microenvironment can be fostered by chromosomal-level alterations (Table 1). Davoli and colleagues [29] discovered that tumor aneuploidy (or somatic copy number alteration, SCNA) is a major determinant of the immunosuppressive tumor microenvironment in a large set of solid tumors, including NSCLC. These authors found that immune evasion markers mainly correlated with chromosome- and chromosome arm-level SCNAs. Tumors with high aneuploidy showed decreased expression of immune-related genes, such as those encoding the TCR complex, the B cell receptor, the cytotoxic function of CD8^+^ cells, the IFN-γ pathway, cytokines, and chemokine genes. Moreover, tumors with high levels of aneuploidy showed reduced CTL/Treg and M1/M2 ratios. In a subsequent study, Kim et al. [30] addressed the role of SCNAs in 248 NSCLC patients treated with immune checkpoint blockade therapy. The level of SCNAs was lower in patients with a partial response than in those with progressive disease/stable disease and in patients with durable clinical benefit compared to those with non-durable clinical benefit.

## 6. Prospects for Overcoming Immune Escape Mechanisms in NSCLC Therapy

Current evidence suggests that some of the immune escape mechanisms that impair NSCLC response to immunotherapy may perhaps be counteracted by the development of appropriate combinatorial treatments. A recent proof of principle study (carried out in colorectal cancer and melanoma pre-clinical models) has shown that loss of *B2M*, a genetic alteration that abrogates MHC class I expression and prevents CD8-mediated tumor recognition, can be overcome by a therapy designed to activated NK cells [47]. In fact, NK cells can be triggered by loss of engagement of inhibitory receptors specific for MHC class I molecules. Administration of the CD122 (IL-2Rβ) IL2 pathway agonist bempegaldesleukin (known as NKTR-214), together with anti-PD-1, to *B2M*-knockout mice, markedly suppressed tumor growth and significantly increased mouse survival [47]. Interestingly, early results of the PIVOT-02 trial [48], based on the association of nivolumab with bempegaldesleukin in patients with multiple tumor types (including NSCLC), has shown a total objective response rate of 59.5%.

Poor antigen presentation, not due to genetic alterations, but to epigenetic silencing of the MHC-I antigen presentation pathway, may in principle be tackled by targeting the functions of the polycomb repressive complex-2 (PRC-2), or by epigenetic drugs such as the DNA-demethylating agents (DNA methyltransferase inhibitors, or DNMTis). Burr et al. [49] have shown that inhibition of EZH2, the catalytic component of PRC2, rescued MHC class I gene transcription in MHC class I-low tumors. Topper et al. [50], in NSCLC models, have shown that combination of the DNMTi azacytidine with histone deacetylase inhibitors (HDACis) can activate a type I IFN transcriptional program leading to the upregulation of HLA class I molecule expression. Different clinical trials based on the association of epigenetic drugs with PD-1 targeted agents are ongoing even in NSCLC [51]. Even defective type I IFN signaling, associated with immunotherapy resistance, may in principle be counteracted by combinatorial treatments. Torrejion et al. [47] have shown, at the pre-clinical level in colorectal cancer and melanoma models, that intra-tumoral administration of a TLR9 agonist could trigger a type I IFN systemic response, overcoming anti-PD-1 resistance.

Future clinical studies in the NSCLC immunotherapy setting will be needed to verify whether these combinatorial approaches can overcome immune escape mechanisms and PD-1 resistance. Nevertheless, available evidence suggests that rationally designed combinations of immune checkpoint blockade, with other anti-tumor agents, may improve treatment of specific NSCLC subsets. Successful examples of such combinatorial treatments are already available and include the IMPower150 study [52] and the KEYNOTE-407 trial [53]. In the IMPower150 trial, significant improvements in PFS and OS have been achieved by the atezolizumab/bevacizumab/chemotherapy combination in metastatic non-squamous NSCLC [52]. In the KEYNOTE-407 trial, significantly longer PFS and OS have been obtained by the association of pembrolizumab and chemotherapy in squamous NSCLC [53].

## 7. Conclusions

The available evidence supports the notion that the activation of multiple immune escape mechanisms is an early event in the progression from pre-malignancy to invasive lung cancer. The development of several immune evasion mechanisms in the precursor lesions, when not counteracted by strong anti-tumor responses, is likely a driving force that promotes the progression of pre-invasive lesions to LUAD and LUSC. In invasive LUAD and LUSC, the immune complexity increases, leading to the generation of a spectrum of lesions characterized by remarkable diversity of the immune structure and by intra-tumor immunological heterogeneity. Furthermore, specific genetic changes and chromosomal alterations, as well as the interaction of neoantigen structure with adaptive immunity, concur to shape LUAD and LUSC immune evolution and to promote the resistance of different NSCLC subsets to immunotherapy targeting immune checkpoints. The development of combinatorial immunotherapy treatments, designed specifically to counteract immune escape mechanisms, appears as a promising and necessary way forward to improve clinical management of NSCLC.

## Figures and Tables

**Table 1:** Immune escape and immunosuppressive mechanisms in pre-invasive lesions, in invasive LUAD and LUSC, and in immunotherapy resistance.

Mechanism	LUAD or Pre-Invasive Lesions	LUSC or Pre-Invasive Lesions	Refs.
**1. Impaired Antigen Presentation**			
-HLA-LOH.	-Progressive increase from AIS/MIA to LUAD.	-Detected in 34% of CIS and 28% of LUSC.	[12,21]
-HLA-LOH.	-Detected in 29% of LUAD, frequently subclonal HLA LOH.	-Detected in 61% of LUSC, frequently subclonal HLA LOH.	[2]
-HLA-LOH.	-Detected in 56% of LUAD.	-Detected in 78% of LUSC.	[22]
-Expression of MHC I/II, B2M, and antigen processing machinery. -NK cell activation and type I IFN production.	-Reduced/defective in subgroup 1 of three early LUAD subsets.		[23]
-Expression of peptide-trimming machinery genes and MHC class II.	-Reduced in subgroup 2 of three early LUAD subsets.		[23]
-Hypermethylation at chromosome 6 HLA region.		-Detected in progressive CIS lesions.	[21]
-Mutations and copy number changes of genes involved in antigen presentation.		-More frequent in progressive CIS lesions.	[21]
**2. Neoantigen Loss/Silencing and Neoantigen Subclonal Structure**			
-Neoantigen promoter hypermethylation.	-Increased in later stage pre-invasive lesions. -Detected in invasive stage.	-Detected in invasive stage.	[11,22]
-High neoantigen subclonal fraction.	-Associated with ICB resistance.		[24]
-Neoantigen loss by immunoediting or through deletion of chromosomal regions and LOH.	-Associated with acquired ICB resistance.	-Associated with acquired ICB resistance.	[25]
**3. Function of Immune-related Genes and Pathways**			
-Genes belonging to the IFN γ and TGF-γ signaling pathways.	-Downregulated in subgroup 2 of three early LUAD subsets.		[23]
-*CD47*, *B7-H3*, *CTLA-4* genes.	-Increased expression from AAH to LUAD.		[14]
-Genes involved in negative regulation of immunity.		-Upregulated in high-grade pre-invasive lesions.	[20]
-Downregulation of *CD137L*.		-Detected in progressive CIS lesions.	[21]
-Expression of genes involved in inflammation, lymphocyte regulation, antigen processing/presentation.		-Decreased in “normal-like” precursor lesions.	[26]
-Genes involved in interferon signaling and antigen presentation.		-Downregulated in the “proliferative” pre-invasive lesions that progress to LUSC.	[26]
**4. Immune Structure of the Tumor Microenvironment**			
-Immune infiltration.	-Lowest in subgroup 1 of three early LUAD subsets.		[23]
-Tregs and immunosuppressive cytokines.	-Higher levels in subset of cold tumors.		[27]
-Tregs, tumor-associated neutrophils.		-Present at higher frequency in LUSC compared to LUAD.	[28]
-TH1 and TH17 and macrophages.	-Present at higher frequency in LUAD compared to LUSC.		[28]
-CD4/CD8 ratio.	-Progressive increase from NL to LUAD.		[12]
-TH1 cytokines.	-Reduced in AIS and MIA vs. AAH.		[12]
-TH2 cytokines.	-Higher in AIS and MIA.		[12]
-CD16^+^ NK cells, CD8^+^ CTLs, CD141^+^ DCs, CD16^+^ monocytes.	-Reduced in LUAD compared to NL.		[3]
-Tregs, CTLs, PD-1^+^ CTLs. PPARγ^hi^CD64^hi^CD14^hi^IL-6^hi^ macrophages.	-Increased in LUAD compared to NL.		[3,27]
-Myeloid cells, neutrophils, macrophages.		-Increase in high-grade dysplasia.	[20]
-Spatial segregation of CD3^+^ cells and epithelial cells.		-Detected in high-grade lesions.	[20]
-Lymphocytes, CD8^+^ cells, and *IL2*, *TNF*, *IL12A*, *IL23A* genes.		-Lower levels in progressive CIS compared to regressive CIS.	[21]
-Immune-cold microenvironment.		-Almost all pre-invasive lesions progress to cancer.	[21]
**5. Genetic Changes Impacting on Immune Escape/Immune Suppression**			
-Chromosome-level and arm-level aneuploidy associated with immunosuppressive microenvironment.	-Associated with ICB resistance.	-Associated with ICB resistance.	[29,30]
-KRAS^mut^/LKB1^mut^ LUAD subset.	-Reduced T cell infiltration and PD-L1 expression. -Defective STING expression and impaired type I IFN pathway. -Downregulation of DC, NK, and macrophage signatures. -Associated with ICB resistance.		[5,31,32]
-*EGFR* mutations and *EML4-ALK* fusion LUAD subsets.	-Immune-cold microenvironment. -Lack of T cell infiltration, constitutive PD-L1 expression. -Associated with ICB resistance.		[4,33,34]

AAH: atypical adenomatous hyperplasia; AIS: adenocarcinoma in situ; MIA: minimally invasive adenocarcinoma; LUAD: lung adenocarcinoma; LUSC: lung squamous cell carcinoma; CIS: carcinoma in situ; HLA LOH: HLA loss of heterozygosity; ICB: immune checkpoint blockade.
